# 3D Exoscopic Surgery (3Des) for Transoral Oropharyngectomy

**DOI:** 10.3389/fonc.2020.00016

**Published:** 2020-01-31

**Authors:** Erika Crosetti, Giulia Arrigoni, Andrea Manca, Alessandra Caracciolo, Ilaria Bertotto, Giovanni Succo

**Affiliations:** ^1^Head and Neck Oncology Service, Candiolo Cancer Institute, FPO - IRCCS, Candiolo, Italy; ^2^Radiology Unit, Surgery Department, Candiolo Cancer Institute, FPO - IRCCS, Candiolo, Italy; ^3^Department of Oncology, University of Turin, Orbassano, Italy

**Keywords:** 3D, transoral surgery, transoral oropharyngectomy, 3D exoscopic surgery, oropharyngeal cancer

## Abstract

Over the past three decades, the incidence of oropharyngeal squamous cell carcinoma has increased, primarily related to the spread of human papillomavirus. Treatment has always been preferentially unimodal (surgery or radiotherapy) for early stage disease and multimodal (surgery with adjuvant therapy or concomitant chemoradiotherapy) for advanced stages. Recently, the surgical approach has gained renewed interest due to the morbidity of non-surgical treatments and also to technical innovations. We have coined the term 3Des (3D exoscope surgery) to describe the use of the 3D Vitom Exoscope System for transoral surgery of oropharyngeal cancers. During the period from June 2017 to May 2018, 10 patients with oropharyngeal cancer were treated by oropharyngeal surgery with the 3Des approach at FPO IRCCS Institute of Candiolo. The aim of the present prospective study was to evaluate the utility of 3Des for the treatment of early-stage oropharyngeal cancer. 3Des could represent a viable alternative to the operating microscope and robotic surgery thanks to its excellent ability to provide 3D visual information, depth of field, magnification, image contrast, color imaging, and low running costs. It promises great utility in the learning process, with the possibility of recording in high definition.

## Introduction

Over the past 30 years, a decline in the overall incidence of head and neck cancer has been observed worldwide due to the reduction in tobacco and alcohol abuse. Conversely, oropharyngeal squamous cell carcinoma (OPSCC), the third most common tumor in the head and neck region, is showing a contrasting trend. In fact, a significantly increasing incidence of OPSCC, particularly tonsillar cancer, has been reported in many countries (1–6). This phenomenon is clearly related to human papillomavirus infection (HPV 16, 18, 33, 35). Patients with HPV-related OPSCCs have a remarkably better prognosis and overall survival rates than patients with HPV-negative tumors (80 vs. 40% 5-year disease-specific survival) (7), but HPV status has not yet had an impact on the choice of treatment. Patients with HPV-related OPSCC might be over-treated, and treatment de-escalation (8) is under investigation in clinical trials, especially in low-risk patients and early-stage disease (9).

The standard treatments for OPSCC are represented by surgery and radiotherapy (RT), alone or in combination, and by chemotherapy (CT) associated with radiotherapy. For early disease stages (T1–T2), conservative transoral surgical procedures are the first choice treatment, aiming to reduce the morbidity and late complications; in particular, transoral laser microsurgery (TLM) and transoral robotic surgery (TORS) have provided multiple demonstrations of good results, both in terms of oncologic and functional outcomes. The latter has proved to be very effective, opening a new era of high-precision minimally invasive transoral surgery, even extending to the deeper neck spaces. One relevant side effect of this technique is the high purchase and management costs, the main reason why it is not yet widely accessible (10); another critical aspect is that not all patients are suitable for a robotic approach.

One of the undoubted advantages of TORS is the three-dimensional endocavitary vision, which allows the surgeon to carry out the procedure with great precision, superior to that of conventional approaches, where only the first surgeon enjoys a good view of the deep surgical field.

The Vitom 3D Exoscopic System is a video telescope operating microscope (VITOM®) introduced by Karl Storz (Tuttlingen, Germany). Applied first in neurosurgery (11–13), urology (14), and gynecologic surgery (11), the use of VITOM® is now beginning to grow in ENT surgery as well. At present, only a few series have been reported in the literature (15, 16).

In our institute, we have used the 3D Vitom Exoscope System for the treatment of tumors of the oropharynx and oral cavity at the early-intermediate stage and for benign pathologies. The aim was to develop and rejuvenate a traditional transoral surgical technique, performed with 3D screen vision and to test its ability in terms of surgical precision and shared surgical vision in comparison to TORS. This preliminary study reports the results of 10 consecutive transoral lateral oropharyngectomies carried out using Vitom 3D, for which we have coined the term 3Des (3D exoscopic surgery), analyzing the efficacy and safety of the procedures.

## Materials and Methods

This was a prospective study using 3Des transoral surgery in patients affected by OPSCC. Ten consecutive patients underwent transoral lateral oropharyngectomy (Huet's procedure) (17) at FPO-IRCCS Institute of Candiolo in the period between June 2017 and May 2018.

All of the procedures were considered to be conventional in terms of technique and indications, in accordance with the current guidelines and therefore also in accordance with the ethical standards of the institutional and/or National Research Committee and with the 1964 Helsinki declaration and its later amendments. Ethical review and approval were not required for this study in accordance with the national and institutional requirements. However, before surgery, every patient signed a consent form for disclosure of appropriate personal data for scientific purposes. Written informed consent was obtained from all of the patients.

All patients underwent the same clinical assessment during the 3 weeks before surgery including: clinical examination, nutritional status evaluation (BMI), biopsy/pathological examination, p16 protein expression on biopsy, maxillofacial and neck MRI/CT scan, and total body PET scan. Two surgeons (G.S. and E.C.) carried out all of the procedures. Demographic data for the whole series are summarized in Table 1.

**Table 1:** Demographic data for the 10 patients in this study.

**N**	**ID**	**Age (years)**	**Sex**	**BMI**	**Pre-treatment**	**Histology**	**Site**	**cTNM (TNM VIII Eds)**	**Inter-incisor distance (cm)**	**Neck dissection**	**Tracheostomy**	**pTNM (TNM VIII Eds)**	**p16**	**Intraoperative length (min) TLO/Total surgery**	**Surgical margins (negative/positive/closed)**	**Hospitalization period (days)**
1	FP	61	Male	29	None	SCC	Tonsil	cT1N1	4.5	Yes	Yes	pT1N1	+	90/310	Negative	8
2	GL	73	Male	26	None	SCC	Tonsil	cT1N3b	3.5	Yes	Yes	pT1N2	+	90/600	Negative	18
3	BPG	78	Male	28	None	SCC	Tonsil	cT1N0	4.5	Yes	No	pTisN0	–	60/180	Negative	6
4	AG	79	Male	25	Surgery + RT	SCC	Tonsil	cT1N0	No teeth	No	No	pT1Nx	–	85/85	Negative	3
5	IL	65	Female	20	Surgery	SCC	Tonsil	rcT1N0	5	Yes	No	rpT1N0	–	120/200	Negative	3
6	AI	71	Female	18	None	SCC	Tonsil	cT1N2a	3.8	Yes	No	pT1N0	+	40/105	Negative	5
7	CS	44	Female	17	None	SCC	Tonsil	cT2N2c	4.5	Yes	Yes	pT2N3b	–	60/585	Negative	12
8	PG	64	Male	27	None	SCC	Tonsil	cT1N1	5	Yes	Yes	pT2N1	+	70/510	Negative	12
9	GG	63	Male	31	None	SCC	Tonsil	cT1N0	5	No	No	pT1Nx	–	60/120	Negative	6
10	RT	76	Male	27	None	SCC	Tonsil	cT2N0	5	Yes	Yes	pT1N0	+	52/110	Negative	6

*SCC, squamous cell carcinoma; TLO, transoral lateral oropharyngectomy*.

### 3D Video Technology

The video telescope operating microscope used in this study was a VITOM® (Karl Storz) exoscope with 0° or 90° camera options. The exoscope is an external 3D optical device, similar to a camera, which has the characteristic of being covered by a sterile sheath making it suitable to be placed proximal to the area where the surgical procedure is taking place. It has built-in fiber optic light transmission, suitable for wipe disinfection. The Vitom 3D system has zoom and focus functions, integrated illumination (upgradeable using a double cable and light source) and horizontal alignment. The camera offers a magnification of 1–2x depending on the working distance, and the size and resolution of the monitor used.

This camera system supports an output signal of 1,920 × 1,080 p, and progressive scanning delivers crystal-clear images with smooth motion, even with rapid movement. The 3-chip sensor technology in the camera head ensures natural color rendition as well as five different types of filter to facilitate vision in every anatomic and lighting condition; these filters also provide greater enhancement of fine irregularities affecting the mucosa. Combining this technology with the high-quality VITOM® system achieves excellent image quality that benefits all surgical disciplines.

In order to improve visualization of the base of tongue and supraglottis, VITOM® can also be replaced by TIPCAM®1 S 3D ORL (Karl Storz), a 3D laparoscopic video endoscope (0° or 30°) with a diameter of 10 mm. TIPCAM® benefits from well-known visualization modes for diagnosis and therapy with clearer differentiation of tissue structures (CLARA, CHROMA, and SPECTRA visualization modes).

Different directions of view are possible depending on the surgical site which is being operated (0° is better for the palate and tonsils; 30° is preferred for the base of the tongue and epiglottis). The camera is connected to a 3D monitor (55″), with a maximum screen resolution of 1,920 × 1,080, and 16:9 image format. 3D passive-polarized glasses, with anti-fog coating, or 3D clip-on glasses, circularly polarized, are worn by all staff in the operating room (OR) to view the monitor. An intuitive control unit with 3D wheel (joystick) is used to control the camera. It has four programmable function keys. A joystick with sterile coating can be used by the surgeons' assistants, or without a coating by other members of the surgical team not working directly in the operating field. The joystick can also be applied to a holding system to be controlled directly by the first surgeon when needed.

A comfortable transoral exposure of the lesion is sought, so as to visualize the lesion and its boundaries completely, and to have sufficient space to move the surgical instruments. Therefore, different types of autostatic retractors can be used. Surgical instruments were at least 24 cm long (from 24 to 30 cm) because of the depth of the structures to be reached. Different kinds of cutting instruments could be used (bipolar forceps, CO_2_ fiber laser, ultrasound tools), and various types of angled tools were also required.

### Surgery

**Figure 1:**
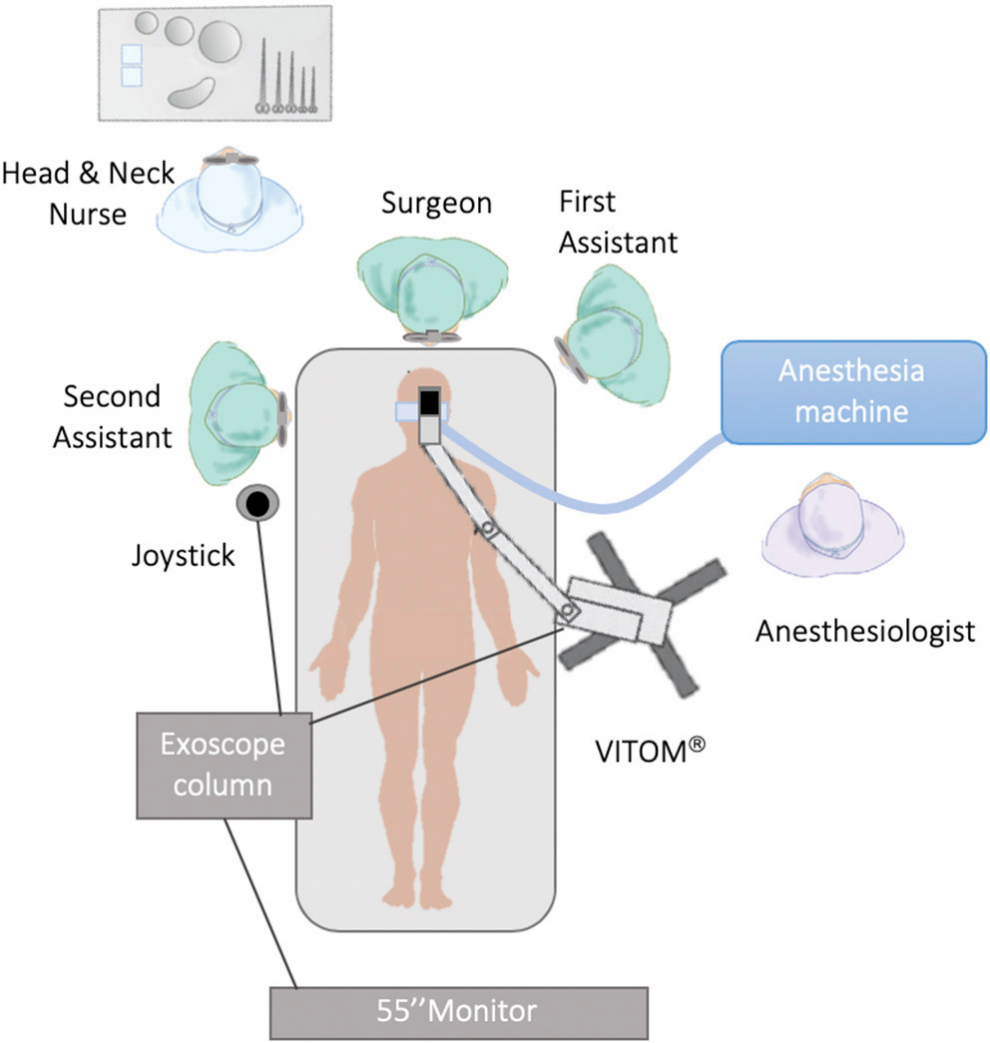
Operating room setting (VITOM® Exoscope).

**Figure 2:**
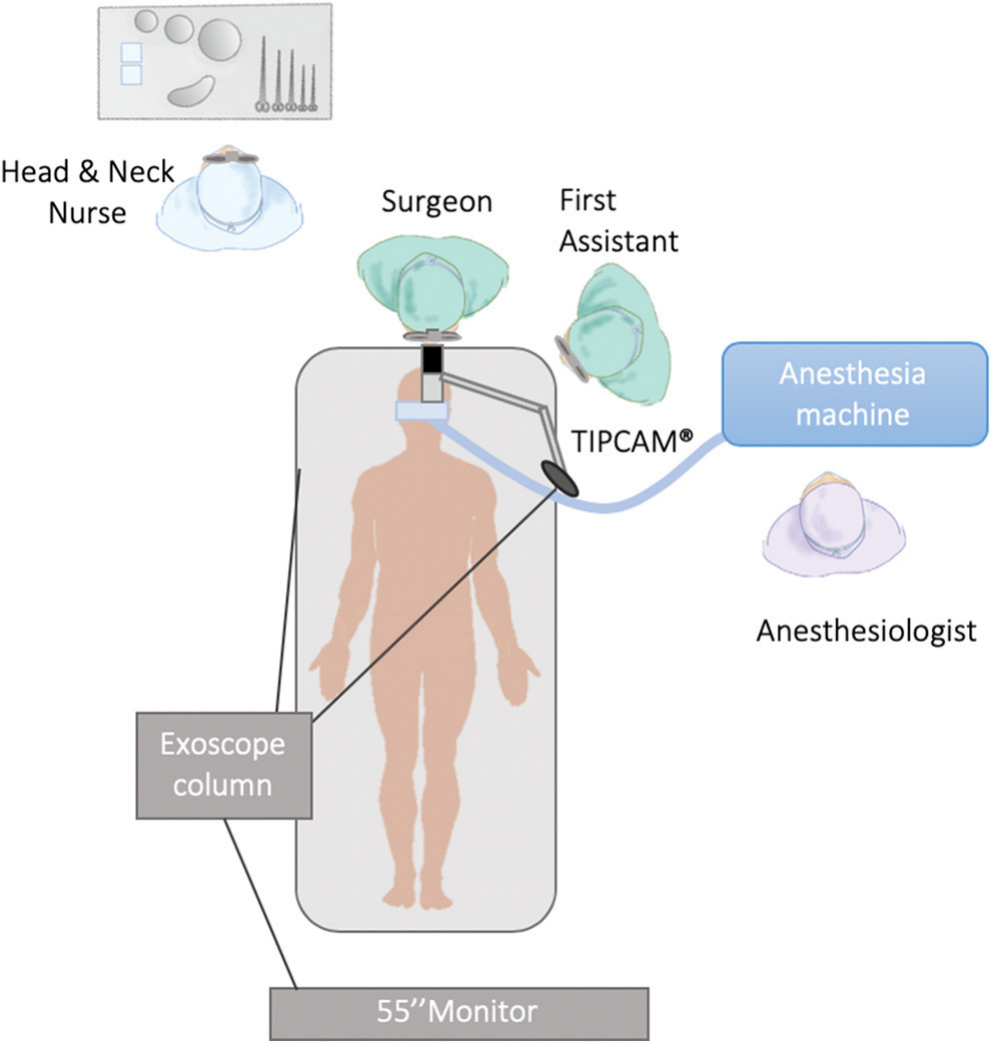
Operating room setting (TIPCAM®).

The patient was placed in the supine position without any interscapular support. The procedures were carried out under general anesthesia performed by nasopharyngeal intubation. In all cases, the 90° VITOM® was assembled on a mechanical holder and with an autostatic arm attached to the bed at a distance ranging between 25 and 50 cm from the surgical field; the system was then covered with a sterile coating (Figures 1, 2). A full HD camera and a light cable were then attached to the VITOM® in the slots provided. Optical magnification is strictly dependent on working distance and monitor resolution. Our main 3D monitor (55″) was placed beside the operating table toward the end of it and directly in front of the first surgeon, while a secondary 3D monitor was oriented in front of the second surgeon. Surgery is more comfortable when performed by three surgeons, but it is always possible for the first or second surgeon to adjust the controller themselves as it is covered with a sterile coating in the surgical field. The surgeon was positioned at the patient's head, facing the monitor. The first surgical assistant sat to the left/right of the first surgeon (depending on the side of the lesion) and assisted in the procedure using retractors and a Yankauer suction tube. The second assistant sat on the opposite side, using the controller (joystick) covered with a sterile coating, and maintained the focus of the camera on the surgical field, adjusting the optical magnification, and applying different camera enhancing tools (SPIES system). The head and neck nurse stood behind the surgeon. All operators wore 3D passive-polarized glasses.

The surgical procedures were carried out according to conventional criteria, following the technique described by Huet (17), which has recently become very popular in the transoral robotic approach. The entire surgical team benefit from the presence of 3D vision during execution of the procedure.

### Statistical Analysis

Means and standard deviations were used to report continuous variables, and frequencies and proportions for categorical variables.

## Results

Ten patients (seven men and three women) underwent 3Des transoral lateral oropharyngectomy. Their median age was 67 years (range 44–79 years). One patient was pre-treated with surgery (simple tonsillectomy for biopsy), and one with surgery and RT [squamous cell carcinoma (SCC) of the tongue].

Interincisor distance measured on average 4.5 cm; one patient was toothless. Eight patients underwent neck dissection; of these, four patients underwent ipsilateral selective neck dissection of levels II-IV, two patients were treated with modified radical neck dissection including level V (one type I and one type III), and two neck dissections were bilateral. Two patients were not subjected to neck dissection.

Five patients underwent temporary tracheostomy. A microvascular reconstruction was performed in four patients, three with a radial forearm free flap, and one with an anterolateral tight free flap. In one patient, reconstruction was with a local Bichat's fat pad. These steps also took advantage of 3Des vision to harvest the flaps. The option to harvest a free flap was determined by the presence of large lymph nodes in continuity with the tumor and requiring careful dissection of the parapharyngeal space and the subsequent requirement to close a wide communication between the pharynx and the neck.

A nasogastric feeding tube was used in 50% of cases and it was removed at a mean of 9 days post-operatively (range 4–14 days). Food intake started on average on the 5th day post-operatively (range from 1 to 14 days post-operatively). Tracheal cannula removal occurred on average on the 3rd post-operative day (range 2–4 days post-operatively).

Post-surgical pain was evaluated with a numerical rating scale (NRS, with range 0–10) (18) and was on average 1.2, with a maximum value of 2. All patients received 1 g of paracetamol intravenously three times a day to relieve pain. None needed supplemental therapy with NSAIDs or opioids.

Overall, no major complications were observed either in the intra-operative or post-operative period. During the post-operative period, one case of subcutaneous emphysema and one submental blood collection were reported, mainly related to tracheostomy and neck dissection.

The average docking time to obtain an adequate operative setting was 7 min. The average intra-operative period was 280 min (including transoral resection, neck dissection, flap harvesting, and tracheostomy), and the average transoral resection period was 73 min.

The average length of hospitalization was 8 days.

The average cost of consumables (VITOM® and joystick sterile coating) per procedure was €62 (€41 and 21, respectively).

The pathological examination identified p16 expression in 5 out 10 patients (50%). Study of the margins of the pathological specimens showed negative margins in all patients.

Three patients underwent adjuvant therapy: two patients received radiotherapy alone (for lymph node metastases), two patients who received the same treatment refused therapy, and one patient was treated with concomitant chemoradiotherapy (for lymph node metastases with extranodal extension, vascular tumor embolization, and perineural spread).

Follow-up at 24 or 30 months showed 9 patients without evident disease. One patient received chemotherapy due to local recurrence and distant metastasis to the lungs, skeleton, and liver; this patient had a p16-negative pT2 pN3b SCC of the left tonsil, and refused adjuvant treatments after surgery.

A total of 41 OR staff (surgeons, anesthesiologists, nurses, students) took part in the surgical procedures. Only two developed discomfort due to the 3D vision: headache and pain in the bridge of the nose. In no case did we have to change the procedure or replace a member of the surgical team.

## Discussion

In the past 10 years, improvements in our knowledge of HPV-related OPSCC, a particular type of tumor showing better prognosis, have triggered the development of less aggressive/invasive therapies; de-intensified treatments and minimally invasive surgical approaches have been steadily rising. The aim is to minimize the morbidity and late-stage complications of the conventional open surgical approach, radiotherapy or concurrent chemoradiation therapies (xerostomia and dysphagia or trismus and osteoradionecrosis) maintaining the same curative effects.

Focusing on transoral surgery, the use of TORS is spreading worldwide with the support of increasing scientific evidence. Thanks to some features of robot-assisted surgery (the DaVinci system from Intuitive Surgical Company and the FLEX® system from Medrobotics) such as 3D intraoral view, articulation of instruments, and one assistant standing in the surgical field, this procedure has been simplified and has therefore contributed to the spread of transoral oropharyngectomy, a minimally invasive surgical technique described by Huet (17) and further developed by Holsinger et al. (19) and Laccourreye et al. (20, 21). At present, this operation is considered the first surgical option for OPSCC in its early stages and has emerged as a potentially effective de-escalation approach.

3D endocavitary vision gives the surgeon immediate feedback of great precision and safety while working next to very delicate and important anatomic structures, and at minimal distance from the lesion. These characteristics are not perceived in conventional transoral approaches. In fact, a possible disadvantage of these procedures is related to the poor precision of the second surgeon's movements due to the reduced visibility of the operating field.

A different surgical option to resect early OPSCC employs an operative microscope coupled to CO_2_ transoral laser microsurgery; the drawbacks of this surgical option are the bulky equipment, difficult docking in narrow anatomical spaces and direct vision guaranteed to the first operator only. In the last few years, several studies have shown that TORS may be an effective alternative to open surgery (22–25). Weinstein et al. reported that the disease control and survival rates as well as the safety of TORS using the Da Vinci surgical system were commensurate with standard treatments (26). A 3-year local control rate of 97% and a disease-specific survival rate of 95% were described by Moore et al. (27).

TORS is very expensive; the cost of a robotic platform is about €3 million, and even maintenance costs are very high (€100,000/year), therefore it is accessible to only a few centers (10, 28, 29). Exposure of the surgical field can be a problem. Holsinger reported significant limitations for the application of TORS in head and neck surgery: first-generation robotic surgical systems with rigid arms were designed for abdomino-pelvic and thoracic surgery; therefore, instrumentation is larger than what would be ideally suited for transoral access to narrow spaces such as the oropharynx and larynx (30). In addition, there are problems related to visual obstruction and instrument collisions as the TORS equipment is very large, and the robotic arms are heavy to move. Moreover, the surgeon's ability to provide good traction is limited by the ability to place routinely only two surgical instruments and the binocular camera. Operating times are prolonged due to lengthy set-up. The surgical technique requires extensive training with complex machinery. Besides, not all patients are suitable for robotic dissection; for example, in the case of non-cancer related trismus, robotic access via the oral cavity is impossible, and dental lesions can frequently occur; cervical spine disease can also interfere with necessary patient positioning during TORS (31). In a study on 31 patients to be treated with TORS, five patients (16%) could not be treated as the surgical field could not be adequately exposed. In a similar study on 23 patients, 6 (16%) were amenable to this technique, and of those, four patients had inadequate exposure of the operating field, and three had a tumor that was too large for the robotic approach (32).

Based on these considerations, and despite having a Da Vinci Xi robotic platform in our institute, we recently decided to study the applicability of the Storz 3D Exoscopic System for oropharyngeal surgery. The aim was to test the usefulness of this sterile optical system in lateral oropharyngectomy. The hypothesis was that the precision and safety of the procedure could be improved significantly by adopting this tool rather than conventional transoral techniques, and with the benefit that it is comparable with TORS and microscopic transoral techniques and with lower costs.

In our experience with 10 cases of oropharyngeal cancer, VITOM proved to be a versatile and compact optical instrument giving an excellent 3D image, without oral cavity involvement. The term 3Des (3D exoscopic surgery) was coined to describe the technique used for transoral surgery with the exoscope system as visual tool.

**Figure 3:**
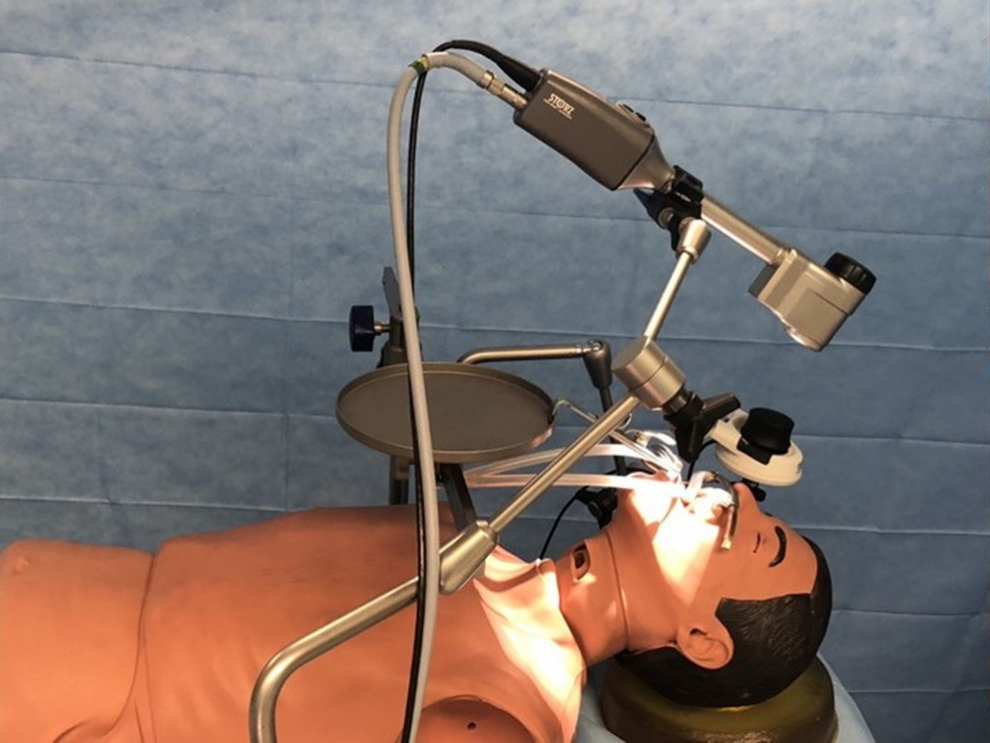
Tests on the mannequin.

The first problem encountered in this study was in identifying the best work setting. After some tests on a mannequin, an ergonomic setting was identified with the optical body assembled on a mechanical holder with three joints and with an extension arm, fixed to the operating table (Figure 3). The same layout, using a different terminal, allows both the exoscope and the 3D laparoscopic video telescope with a diameter of 10 mm to be set up, and it is useful to extend the angled vision to the base of the tongue and to the entrance of the piriform sinus. However, using this holder, the exoscope is not easy to position and move; at present, this is a weak point of the technique that makes maneuvering and setting up the optimal operative position less fluid.

An advantage of the system is linked to anchoring of the instrument to the operating table with consequent absence of variation of the optical axis with movements of the same. Further research must be oriented to the development of an electromagnetic holder that makes the positioning of the exoscope precise and sensitive.

For execution of lateral oropharyngectomy, the head of the Vitom is positioned about 35–40 cm from the patient's mouth, along the visual axis between the surgeon's eye and the operative target, and which replaces the eyes of the operating team members. This provides a shared and identical vision for all of the procedure participants looking directly at two 3D monitors (main and accessory), placed at 90° between them, at the height of the eyes of the surgeons and at the feet of the patient. The characteristics of the 3Des images can be considered very comparable to those of the operating microscope and the 3D optics of Da Vinci in TORS, with its excellent ability to provide 3D visual information which is used to interactively maneuver the exoscope camera. Other advantages are its depth of field, magnification, and image contrast and color, allowing direct manipulation of the images of anatomic structures. The most advantageous aspects are represented by the magnification of the anatomic details, for example, the vascularization and irregularities of the mucosa that become perfectly visible. The 3Des provides an ample working space and is extremely useful for training and educational purposes. Both images and video sequences can be stored digitally (15).

Usage is comfortable for the operator, who can choose to stay in a sitting or standing position, having the screen in front at the same height. Surgery performed with a 3D screen is not bothersome for operators, even for longer procedures, as long as the screen is placed frontally, and at the same height as the operators' eyes. For execution of all of the procedures, conventional surgical instruments have been used (no requirement to buy other specific instrumentation), and this, in terms of immediacy, simplicity of use and low cost, is undoubtedly an advantage. Other hemostatic tools can be used (Focus, Ligasure, Thunderbeat, flexible CO_2_ fiber laser, etc.) with safety deriving from complete visual control.

We have applied the 3Des approach for transoral resection of OPSCC in 10 cases, with or without neck dissection. Five cases (salvage surgery, loco-regional extension) underwent reconstruction: three with a forearm free flap, and one with an anterolateral tight free flap because of a concomitant oral cavity lesion. Another patient underwent reconstruction with a local Bichat's fat pad. These reconstructive steps also took advantage of 3Des by VITOM in harvesting and suturing the flap, since the vision that the VITOM 3D allows facilitates transoral suturing of the flap to the mucosa; the combination of perfect vision and use of barbed suture resulted useful to reduce the fistula rate. Surgical procedures for the transoral approach enjoyed the same benefits as provided by TORS, in terms of lower morbidity, fewer complications, and faster local healing and rehabilitation. Hospitalization times were short (8 days) compared, in our experience, to about 15 days required after an open approach, and we encountered no complications (apart from two cases mainly due to tracheostomy and neck dissection).

The surgeons found that it was very easy to familiarize themselves with 3Des and incorporate it into the surgical routine, and they did not find its use unduly tiring on their eyes. Surgical field depth achieved is satisfactory as 6–7 cm could be reached if the exoscope is held at about 40 cm distance. The platform's optical ergonomics are adequate, the exoscope/holder/camera control wheel (joystick) are completely covered with a sterile coating, and camera control is available in the surgical field. The 3Des approach has several advantages. First, the possibility to approach surgery transorally with indirect but straight visualization, for the whole surgical team, affording all of the members of the team the opportunity to work with great surgical precision. The indirect testimony of this fact is represented by the absence of major complications during surgery and in the immediate post-operative period, and by the status of the surgical margins on the specimens: 10/10 negative (>3 mm).

3Des provides the benefits of great utility in the learning process, especially for residents, fellows, students, and OR staff, thanks to the same shared visual experience being available to every operator, and always with wide high-resolution screens. First, in the cadaver lab and then in the OR with the proctor, 3Des can lead the learners' surgical maneuvers, and trainees may gain confidence with the anatomic structures and microsurgical techniques watching directly on the 3D screen. The inside-out anatomy study, and the indispensable knowledge for surgeons who approach transoral surgery of the oropharynx are facilitated by the 3Des approach, both for the fidelity of vision as well as for the logistics that makes it more easily transportable in the cadaver lab than the robotic platform. Moreover, the possibility to record in high definition enables the surgeons to share videos for didactic sessions, meetings and surgical technique courses.

Set-up of 3Des is easy and intuitive. Thanks to our previous experience with TORS, we can assert that 3Des and TORS enjoy similar vision. 3Des allows the direct maneuverability of instruments providing a tactile sensibility, impossible to achieve when operating with TORS. Furthermore, the 3D exoscope allows a careful endoscopic work-up useful to check the correct surgical field exposure and therefore the possibility to carry out a good and safe robotic resection. A well-executed work-up can also save time during setting up for robotic surgery, for example, by assessing beforehand which self-retaining retractor to use.

At present, in a health policy aimed at reducing costs, it is difficult to procure up-to-date technologies. The whole cost of the exoscopic platform is similar to that of an operating microscope with an electromagnetic brake stand and is about 10 times lower than that of a Da Vinci robotic platform. The cost of disposables for each surgical procedure is about €40–60, composed of two sterile sheaths for the holder and the controller chamber; even the price of maintenance is considerably lower.

The current drawbacks can be represented by the mechanical holder arm that is not always comfortable to move during surgery, and the necessity to wear 3D glasses for a prolonged period that can lead to headaches and nasal pain (only in two subjects out of 41 in our experience).

Although our sample is small, our initial feeling on the 3Des approach for transoral resection of OPSCC by VITOM was good. This device is comfortable to use, allows good magnification of the anatomic details in 3D vision, and guarantees “precision” surgery. The procedures enjoy similar benefits as provided by TORS in terms of precision, oncological radicality, lower morbidity, fewer complications, and faster local healing and rehabilitation.

With the introduction of any new surgical approach, it is common to face difficulties achieving the optimum layout of the operating room, and the most favorable position for the exoscope/holder/camera control wheel (joystick) in the surgical field. In any case, however, the level of fine operativity achievable by TORS in the parapharyngeal space dissection (retropharyngeal lymph nodes dissection) is not yet reachable by 3Des approach, due to the absence of *ad hoc* designed surgical instruments, as well as a robotic holder for VITOM.

## Conclusions

In the wide variety of OPSCC treatments, minimally invasive transoral surgery is able to provide a “custom tailored” approach, giving an excellent chance of recovery combined with a satisfactory quality of life for the patient.

After this preliminary study, to our knowledge the first published in the literature, it is possible to state that the 3Des approach can be added to the other well-known strategies for transoral resection of OPSCC, and can also have immediate and straightforward application in non-oncologic surgical procedures (tonsillectomy—lateral pharyngoplasty, etc.).

The exoscopic platform system has been improved thanks to the development of 10 mm diameter 3D optics (0–30°) useful to treat those cancers in the tonsillar region toward the base of the tongue and vallecula.

3Des has other useful applications in head and neck surgery. In the reconstructive field, it can be useful in free flap insetting in the posterior oral cavity/oropharynx without opening the mandible, and in performing vessel anastomosis in place of the surgical microscope. Thanks to the introduction of 3Des, a substantial number of transoral lateral oropharyngectomies, which would otherwise have been performed by TORS, can now be performed with this less expensive but equally precise and effective technique in the minimally invasive treatment of early OPSCC.

Future applications could include the use of 3Des to aid in retroauricular neck dissection with conventional techniques.

## Data Availability Statement

The datasets generated for this study are available on request to the corresponding author.

## Ethics Statement

Ethical review and approval was not required for this study in accordance with the national and institutional requirements. However, every patient preoperatively signed a consent form for disclosure of privacy in managing personal data for scientific purposes. A written informed consent was obtained from all the patients.

## Author Contributions

GS: conception and design of the study, surgeon, editing the manuscript. EC: conception and design of the study, surgeon, writing and editing the manuscript. GA, AM, and AC: data collection, writing and editing the manuscript. IB: radiologist who performed pre-operative imaging, writing and editing the manuscript.

### Conflict of Interest

The authors declare that the research was conducted in the absence of any commercial or financial relationships that could be construed as a potential conflict of interest.
